# Learning person-centred consultation skills in clinical medicine: A randomised controlled case study

**DOI:** 10.4102/safp.v62i1.5109

**Published:** 2020-07-06

**Authors:** Jakobus M. Louw, Johannes F.M. Hugo

**Affiliations:** 1 Department of Family Medicine, Faculty of Health Sciences, University of Pretoria, Pretoria, South Africa

**Keywords:** person-centred practice, collaboration, facilitation, consultation skill, quality improvement, clinical associate education

## Abstract

**Background:**

Training institutions need to ensure that healthcare students learn the skills to conduct person-centred consultations. We studied changes in person-centred practice over time following a quality improvement (QI) intervention among Bachelor of Clinical Medical Practice undergraduate students.

**Methods:**

Students were randomised to intervention and control groups. The intervention group received training and did a QI cycle on their own consultation skills. Consultations with simulated patients were recorded during structured clinical examinations in June (baseline) and November (post-intervention) 2015.

**Results:**

Matched consultations for 64 students were analysed. The total SEGUE (Set the stage, Elicit information, Give information, Understand the patient’s perspective and End the encounter scores) were significantly higher in the final assessment compared to baseline for both the whole group and the intervention group (*p* = 0.005 and 0.015, respectively). The improvement did not differ significantly between intervention and control groups (*p* = 0.778). Third-year students improved significantly more than second years (*p* = 0.007).

**Conclusion:**

The person-centred practice (including collaboration) of clinical associate students did improve over the period studied. The results show that students’ learning of person-centred practice also happened in ways other than through the QI intervention. There is a need to develop students’ collaborative skills during the medical consultation.

## Introduction

Person-centred practice can be described as practice where clinicians and patients collaborate on the basis of a holistic understanding of the patient and his or her health needs in the milieu of a therapeutic alliance between patient and clinician.^1^ Ethically, it is driven by the obligations to apply the principles of beneficence and autonomy in healthcare.^2^ Practically, it has benefits for patients, clinicians and the healthcare system.^3,4,5,6,7^ Benefits include increased patient^6,8,9^ and clinician^8,10,11^ satisfaction, improved adherence to management plans^8,12,13^ and more efficient care being delivered.^14^

Collaboration, including shared decision-making, is regarded as quintessential person-centred practice.^15^ As articulated in the Salzburg Global Seminar statement on shared decision-making,^16^ this means recognising the ethical imperative to share important decisions with patients, stimulating a two-way flow of information and encouraging patients to ask questions, explain their circumstances and express their personal preferences.

Given the importance of and the need for clinicians to have person-centred practice skills, every institution training healthcare professionals needs to ensure that students learn person-centred practice, including the skills needed to involve the patient in understanding the problem, share decision-making and negotiate as part of collaboration in the medical consultation.^17,18^ They need to be guided in their attitudes to show empathy, compassion and caring, and they need to become proficient in communication, reflection, negotiation, collaboration, mindfulness and other critical ‘soft skills’. Among the ways that these skills and attitudes can be learned are through role-plays of simulated consultations,^17,19^ review of recorded consultations,^20^ feedback on directly observed consultations,^10,21,22^ patient feedback,^23^ observing and working with positive role models,^24,25^ reflective practices,^26^ small group discussions with role models, student-centred community-based learning,^27,28^ patient-centred learning (learning from real patients)^29^ and mindfulness training.^26,30^

Despite the numerous methods suggested for improving skills, a recent Cochrane’s review^31^ could not find any good evidence for the effectiveness of any interventions to increase the use of shared decision-making by healthcare professionals. There is therefore a need to develop training methods so that patients can experience ‘nothing about me without me’.^15^

The study reported here aimed to measure changes in person-centred practice over time following a quality improvement (QI) intervention for learning person-centred consultation skills among Bachelor of Clinical Medical Practice (BCMP) undergraduate students. As graduates, clinical associates qualify to practise as mid-level medical professionals who perform many of the tasks medical doctors usually perform, similar to the physician assistant or clinical officer professions in other countries such as Malawi, Tanzania and the United States.^32,33^

## Methods

In this case study, an intervention group of second- and third-year students was randomly selected through clustered sampling with the remaining students serving as controls.

### Study population

All second- and third-year BCMP students at the University of Pretoria in 2015 were eligible. They were learning in 19 different clinical learning centres (CLCs) based at public hospitals in the Gauteng, Mpumalanga and KwaZulu-Natal provinces.

### Sampling

Five of the CLCs had both second- and third-year students, while seven had only second-year and seven only third-year students. From each of these three clusters of CLCs, three CLCs were randomly selected. After randomisation, the three students at one of the selected second-year CLCs were moved individually to three other CLCs (two intervention and one control CLC). The remaining eight selected CLCs received the learning intervention, while the students at the other 10 CLCs served as controls.

Information on the study was provided to all BCMP II and III students and they indicated their consent electronically on the computer-based testing system at the University of Pretoria.

To be included second- or third-year BCMP students had to complete both a baseline and final consultation assessment and consent to audio or video recording of the consultations.

Because of equipment malfunction on the first day of assessment recording, several third-year students were also excluded.

**FIGURE 1:**
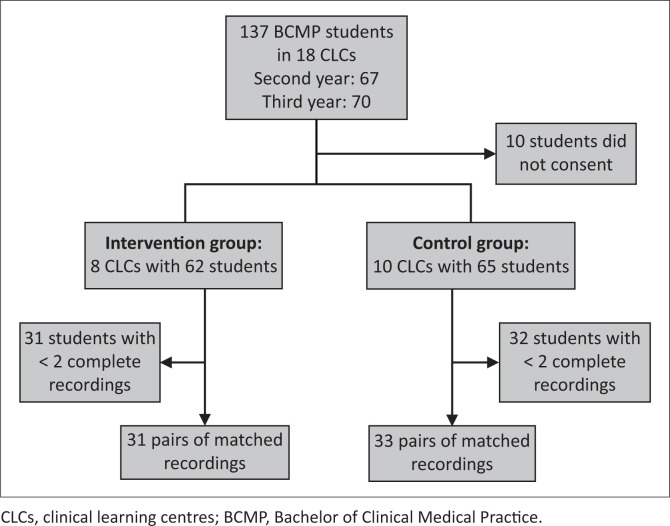
Sampling framework for recordings analysed.

A total of 64 sets of recordings of baseline and final consultations were available for analysis (Figure 1).

### Intervention

The researcher sent emails with reading material and detailed instructions for the intervention to the students in the intervention CLCs. During subsequent site visits to intervention CLCs, the intervention was explained. Role-play was used to demonstrate how to observe a consultation and give appropriate feedback. Any questions were clarified and students were encouraged to engage with the QI process.

The students in the intervention CLCs were expected to:

- form a team of two to four fellow students in the same year group to work together to improve their consultation skills
- read and reflect on two articles describing the medical consultation^34,35^
- study four consultation assessment tools: Kalamazoo Essential Elements Communication Checklist (adapted) – KEECC(A),^36^ Consultation Peer Assessment Tool (as adapted for students at the University of Pretoria), CARE Patient Feedback Measure^37^ and Patient Enablement Instrument^38^
- measure their current consultation practice by assessing each other’s consultations with the tools provided. Consultations could be video-recorded, audio-recorded and/or observed in person. Then they were required to give feedback to each other based on the tools and to reflect on patients’ perceptions of their consultations as recorded in the tools. The final measurement was a self-assessment using one or two of the tools
- plan and implement measures to improve their own consultations
- repeat the measurements of their consultation practice
- reflect on changes in their performance and submit a report on this QI process.

Fidelity of implementation was reviewed using the conceptual framework proposed by researchers at the University of Sheffield.^39^

### Measurements

During the objective structured clinical examinations (OSCEs) at the end of each semester (June and November 2015), consultation skills were evaluated. All students (intervention and control groups) conducted a 13-min consultation with a simulated patient based on one of five standard scenarios. The scenarios were allocated according to the particular clinical rotations the specific student group did in the preceding semester. Students had no access to the scenarios before the examination and no student had the same scenario in the baseline and final measurements. Only one of the five scenarios was used in both the baseline and final evaluations. The consultations were video- and/or audio-recorded in line with the consent provided by the student. For the purpose of this study, only audio recordings were coded for person centeredness. Where only a video recording was available, it was converted to audio before scoring. The SEGUE (Set the stage, Elicit information, Give information, Understand the patient’s perspective and end the Encounter) framework was selected as the preferred measurement tool based on a systematic review.^40^ It consists of 32 tasks, each of which can receive a code of ‘Yes’, ‘No’ or ‘Not applicable’ (Appendix 1).

Two qualified clinical associates received 4 hours of training in the use of the SEGUE measurement tool. Every audio recording was randomly assigned to one of them. They were blinded as to the pre- or post-intervention status of each recording and to the group (intervention or control). Each coder was assigned equal numbers of intervention and control group recordings. The baseline and final recordings of each student were coded by the same person.

Task 5 (Maintain patient’s privacy) and task 21 (Acknowledge waiting time) were not applicable in the context of the OSCE and therefore not coded.

Statistical analyses were conducted on the scores using the IBM Statistical Package for Social Sciences (SPSS) statistics version 25 software. Statisticians from both the Faculty of Health Sciences and the internal consultation service of the University of Pretoria’s Department of Statistics were involved in data analysis. Effect size was measured with Cohen’s *d*, and *p* < 0.05 was regarded as statistically significant. Bonferroni adjustment was applied for multiple comparisons.

Intra- and inter-rater reliabilities were measured by assigning 24 recordings to both coders and by re-allocating at least 22 previously coded recordings under a new random number to the same coder later in the process. The mean of kappa (measure of agreement) calculated for intra-rater reliability across the 30 tasks was 0.9 for coder A and 0.82 for coder B. The mean kappa for inter-rater reliability over 22 tasks was lower at 0.54. (For eight tasks, inter-rater agreement could not be calculated because of a lack of variability in at least one measurement).

Considering the nature of medical consultations, the SEGUE framework contains a mix of tasks measuring various communication abilities. Internal consistency is therefore not regarded as an appropriate criterion for the SEGUE framework.^41^

To summarise the degree to which person-centred communication tasks were accomplished, total SEGUE scores were calculated by assigning a value of 1 to each ‘yes’ and 0 to each ‘no’ and summing the scores for each consultation as performed in previous research.^41^

Results were first compared using paired samples *t*-tests. Multivariate regression was employed to model the final total SEGUE scores against group (intervention group vs. control group), year of study (second vs. third) and gender (male vs. female), taking into account the interactions between gender and year of study and between gender and group, adjusted for the baseline total SEGUE scores.

To evaluate the possible effect of variable implementation of the intervention by students in the intervention group, the intervention group results were divided into those who fully implemented the intervention (submitted written reports), those who implemented partially (did not submit written reports) and those who did not implement the intervention.

Totals for each of the five components of the SEGUE framework were calculated and analysed as subscales. The seven tasks under ‘New or modified treatment or prevention plan’ were analysed as part of the ‘End the encounter’ subscale.

### Ethical consideration

The study was granted ethical clearance from the Research Ethics Committee of the Faculty of Health Sciences, University of Pretoria. The QI intervention was specifically approved as an amendment to the original protocol (128/2013). No patient identifying data were collected. For the statistical analysis, student data were arranged by numbers to ensure confidentiality.

## Results

The demographic characteristics of the study population and participants are presented in Table 1.

**TABLE 1:** Demographic data.

Characteristic	Study population		Sample			
			Intervention		Control	
	*n*	%	*n*	%	*n*	%
**Female**	69	50	10	32	21	64
**Male**	68	50	21	68	12	36
**Year of study**						
Second year	67	49	22	71	21	64
Third year	70	51	9	29	12	36
**Average age**	22.9 years		23 years		21.9 years	
**Age distribution**						
< 20	8	6	1	3	4	12
20–22	79	58	15	48	22	67
23–25	34	25	12	39	6	18
26–28	10	7	2	6	0	
> 28	6	4	1	3	1	3

**Total**	**137**	**-**	**31**	**-**	**33**	**-**

The 25 missing data points were because of poor quality of audio recordings. The SEGUE total scores and subscale scores were adjusted for missing values before analysis.

### Fidelity of implementation

Only 5 of 62 intervention group students did not attend the training. Matched recordings of three of these five were included in the intervention group for analysis.

Of the 31 students analysed in the intervention group, eight did not implement the QI cycle. However, their results were analysed with the intervention group (intention-to-treat analysis). Only 12 students in the intervention group submitted reflective reports.

### Results of total SEGUE scores

The total SEGUE scores of the 64 pairs of matched student consultations showed a significant improvement over the 5 months studied (Table 2). Although the intervention group improved significantly from the baseline to the final assessment, this improvement was not significantly better than for the control group. The control group’s scores did not improve significantly.

**FIGURE 2:**
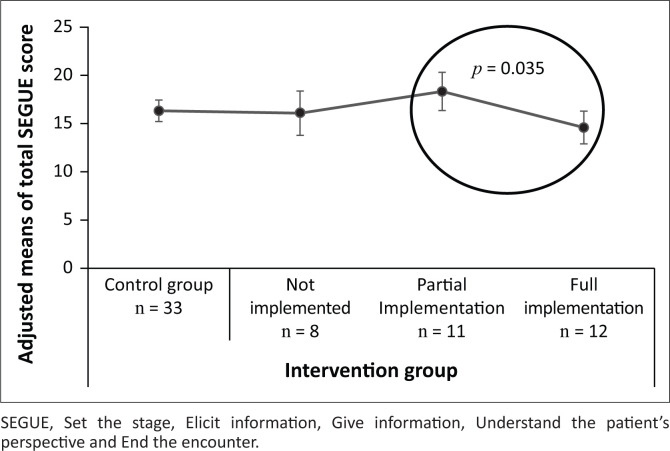
Comparison of adjusted means according to degree of implementation of the intervention with 95% confidence interval.

**TABLE 2:** Comparison of means of total SEGUE scores.

Group	Unadjusted means of total SEGUE scores (Max = 30)						Adjusted mean‡	*p* §
	Baseline	SD	Final	SD	*p* †	Effect size: Cohen’s *d*		
All (*n* = 64)	14.9	3.20	16.3	3.01	0.005*	0.46	-	-
Intervention group (*n* = 31)	14.9	2.50	16.6	3.40	0.015*	0.56	16.28	0.778
Control group (*n* = 33)	14.8	3.77	16.0	2.61	0.118	0.37	16.07	
Male students (*n* = 33)	15.0	2.62	16.9	3.34	0.010*	0.59	16.89	0.070
Female students (*n* = 31)	14.7	3.51	15.6	2.50	0.191	0.31	15.53	
Second years (*n* = 43)	14.9	3.00	15.7	3.04	0.216	0.24	15.66	0.007*
Third years (*n* = 21)	14.7	3.64	17.5	2.60	0.003*	0.89	17.76	

SD, standard deviation; SEGUE, Set the stage, Elicit information, Give information, Understand the patient’s perspective and End the encounter.

* , Significant at the *p* < 0.05 level.

† , Two tailed paired samples *t*-test.

‡ , Mean in final assessment adjusted for baseline.

§ , Multivariate regression analysis.

The multivariate regression model demonstrated that third-year students improved significantly more than second-year students, but the difference in improvement in scores between male and female students was not significant (Table 2).

Figure 2 compares the means of the total SEGUE scores in the final assessment of the control group with the subgroups in the intervention group after adjustment for the baseline scores. The subgroup of the intervention group students who implemented the intervention partially had the highest adjusted means (signifying that they had the best improvement), whereas those who did implement the intervention completely had the least improvement. The difference between these groups was significant (*p* = 0.035).

Excluding the non-implementing subgroup from the analysis (per-protocol analysis) did not affect the significance of the difference between the intervention and control groups.

Neither the relationship between student age and total SEGUE scores, nor between age and changes in the total SEGUE scores were statistically significant.

When students interviewed simulated patients of a different gender (discordant) than their own in the final assessment OSCE, they achieved a significantly higher total SEGUE score. The mean difference was 2.34 (95% CI, 0.9–3.7) and *p* = 0.002 (Cohen’s *d* = 0.82). However, gender discordance did not have any effect in the baseline scores. The simulated patients’ gender did not have any significant effects independently.

### Results of analysis in subscales

**FIGURE 3:**
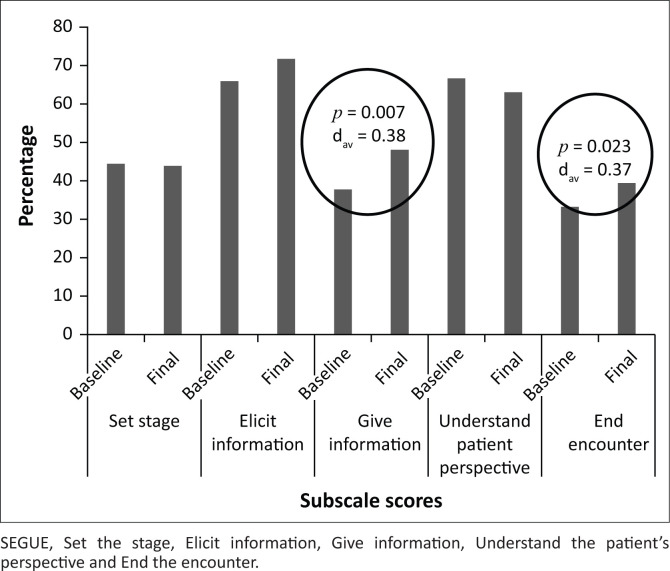
Changes in SEGUE subscale scores between baseline and final assessments.

The ‘Give Information’ and ‘End Encounter’ SEGUE subscales relate closely to collaboration in the consultation. These had lower scores than the other three subscales but improved significantly over the 5 months studied. Changes in the other three subscales were not significant (Figure 3).

Third-year students improved significantly more than second-year students in the ‘Elicit information’ subscale (*p* = 0.020; Cohen’s *d* = 0.59, 95% CI, -5.85 to 6.70).

There was a significant, moderate degree of positive correlation between the improvement in the ‘Elicit information’ and the ‘End encounter’ subscales (Pearson’s correlation coefficient = 0.321, *p* = 0.01).

There were no statistically significant relationships between student age and any of the subscale scores nor with any changes in the subscale scores.

### Results of analysis of specific tasks

In comparing the improvement in specific tasks between intervention versus control groups, third- versus second-year students and male versus female students, differences were not significant (two-sided Fischer’s exact test with Bonferroni adjustment).

## Discussion

This study evaluated the actual behaviour of students in the medical consultation and not merely self-reported attitudes regarding person centeredness. We tested whether a QI intervention implemented by students themselves would improve their person-centred practice. The study did not demonstrate a statistically significant effect of the intervention when comparing the intervention group to the control group. This may be because of the exposure of both the control and intervention groups to other avenues of learning person-centred practice such as the role models (healthcare practitioners)^24,25^ they worked with, small group discussions and role-plays.^17,19^ It is also possible that motivated, self-directed students in the control group used the information provided during the informed consent process to learn person-centred practice.^42^ Students were not closely supervised during the intervention and as it would not directly affect their marks, some students probably lacked motivation to put effort into the QI. Even so, analysis of the results according to the assumed degree of implementation of the intervention did not reveal a dose–response effect. Why the 12 students who implemented the intervention completely had the lowest total adjusted SEGUE scores (Figure 2) is not clear. This result could suggest that reporting on learning does not correlate with actual learning of person-centred practice. Equally, it may be the consequence of other, unaccounted for variations in implementation.

The effect size of the improvement measured in the group as a whole can be regarded as educationally significant though not necessarily practically or clinically relevant.^43,44,45^

Previous research in the United States could not find a difference between the total SEGUE scores for first-year family medicine residents compared to third-year residents.^46^ In our study, the baseline measurements of second- and third-year students did not differ significantly. However, third-year students improved significantly more than second years over the period studied resulting in significantly higher scores in the final assessment. The effect size of this difference in improvement was moderate to large (Cohen’s *d* = 0.76) and, therefore, both practically and educationally meaningful.^43,44,45^ The difference can be attributed to third years improving significantly more in the ‘elicit information’ subscale and to some extend in the ‘end encounter’ subscale, perhaps suggesting a more mature approach to the consultation.

When trying to learn both clinical reasoning and person-centred consultation skills simultaneously, students can feel overwhelmed.^47^ Consultations with real patients trigger empathy and a sense of responsibility in students. Even so, feeling primarily responsible for their patient’s medical decisions, students tend to prioritise clinical reasoning.^47^ The greater improvement by third-year BCMP students, as compared to those in second year, can thus be understood in terms of cognitive load theory. Second-year students could not learn complex consultation skills as well because they have less information organised in cognitive frameworks or concepts (automated schemas) to help them organise and interpret new information, compared to third years who have already internalised more skills in schemas and thus can learn new skills more efficiently without overloading their working memory.^48,49^ This demonstrates the important role of time that goes beyond spacing effects in acquiring person-centred consultation skills. Students need time to develop from clinical knowledge to critical thinking and decision-making skills.^50^ In addition, third-year (final) students could be more focused and motivated to learn because they would soon have to pass final examinations and then enter practice as clinical associates.

Intra- and inter-rater reliabilities were lower than what has been reported in the literature,^41^ but the means of total and subscale scores did not differ significantly between the coders. Poor inter-rater reliability is a common problem. A recent systematic review reported it to be poor in six of seven coding schemes for which they could find valid measurements.^51^

It is difficult to understand the effect of gender discordance in the final assessment in light of the absence of such effect in the baseline assessment.

As shared decision-making – or collaboration with the patient – is crucial for person-centred practice,^15^ we have to evaluate if and how clinical associate students learn to collaborate with patients.

For medical students, lower scores for ‘Ending the session by summarising and clarifying the plan’ than for other subscales have been reported.^21^ Similarly, BCMP students had their lowest scores in the ‘End the encounter’ subscale. However, it is encouraging to find an increase in this subscale over the period studied – especially among third-year students. Its positive correlation with the ‘Elicit information’ subscale has logic: a clinician cannot collaborate with a patient without a good holistic understanding of the patient. The observation that third-year students improved significantly more than second years in the ‘elicit information’ subscale shows that learning of biomedical consultation skills accelerates towards the end of the course.

The data analysed in this study concur with the literature that students are more likely to implement ‘caring’ aspects of person-centred practice while struggling to consistently share power or collaborate with patients. As stated elsewhere: ‘Although talk about patient-centred care is ubiquitous in modern healthcare, one of the greatest challenges of turning the rhetoric into reality continues to be routinely engaging patients in decision making’.^15^

The finding that male students had higher total SEGUE scores than female students was surprising and contrasts with most other reports of measures of person centeredness where female healthcare providers are usually more person-centred than their male counterparts.^21,52,53^ In the intention-to-treat analysis, the effect of student gender did not reach statistical significance but it warrants further quantitative and qualitative research to confirm or refute it and to understand the possible reasons for it.

## Limitations

There are a number of limitations to the study, including the fact that measurements in this study relied on simulated consultations, and thus, results may not be generalisable to clinical practice with real patients.

All aspects of the students’ implementation of the QI were not documented. We can therefore not be sure about the effect of variable implementation of the intervention on the results.

The analysis did not control for other possible methods of learning person-centred practice neither for the possibility of partial implementation of the intervention by the control group.

The smaller than intended sample size limited the statistical power to detect differences. With a larger sample, the difference between male and female students may have reached statistical significance in the intention-to-treat analysis.

## Conclusion

Person-centred practice of second- and third-year clinical associate students did improve marginally over the 5-month period studied, although the study intervention did not contribute significantly to this improvement. The fact that person-centred practice improved significantly more among third-year students’ suggests that these skills are most effectively learned in the last part of the course.

This said, the measurement of person centeredness in the medical consultation remains difficult.^54,55^ Further research should explore comparisons with locally developed measurement tools and/or the appropriate adaptation of existing international tools. Also, the quality and extent of the implementation of any intervention needs to be monitored and effectively documented to derive definitive conclusions on its effectiveness.

## Recommendations

Clinical associate students learn person-centred practice through a range of activities. Further research is indicated to identify and measure sources of such learning.

Further studies are needed to understand the effect of gender concordance versus discordance between student and simulated patient in consultation OSCE stations.

## Appendix 1: The SEGUE framework.

**Table:**

	Yes/No/n/a
**Set the stage**	
1. Greet the patient appropriately	
2. Establish the reason for the visit:	
3. Outline agenda for visit (e.g. issues, sequence)	
4. Make a personal connection during visit (e.g. go beyond medical issues at hand)	
5. Maintain patient’s privacy (e.g. knock, close door)	
**Elicit information**	
6. Elicit the patient’s view of health problem and/or progress	
7. Explore physical and physiological factors (signs and symptoms)	
8. Explore psychosocial and emotional factors (e.g. living situation, family relations, stress, work)	
9. Discuss antecedent treatments (e.g. self-care, last visit, other medical care)	
10. Discuss how the health problem affects the patient’s life (e.g. quality of life)	
11. Discuss lifestyle issues or prevention strategies (e.g. health risks)	
12. Avoid directive or leading questions	
13. Give the patient the opportunity or time to talk (e.g. don’t interrupt)	
14. Listen. Give the patient your undivided attention (e.g. face patient, give feedback)	
15. Check or clarify information (e.g. recap, ask ‘how much is not much’)	
**Give information**	
16. Explain rationale for diagnostic procedures (e.g. exam, tests)	
17. Teach patient about his or her own body and situation (e.g. provide feedback and explanations)	
18. Encourage patient to ask questions or check his or her understanding	
19. Adapt to patient’s level of understanding (e.g. avoid or explain jargon)	
**Understand the patient’s perspective**	
20. Acknowledge the patient’s accomplishments or progress or challenges	
21. Acknowledge waiting time	
22. Express caring, concern, empathy	
23. Maintain a respectful tone	
**End the encounter**	
24. Ask if there is anything else patient would like to discuss	
25. Review next steps with patient	
**If you suggested a new or modified treatment or prevention plan**	
26. Discuss patient’s interest or expectation or goal for the plan	
27. Involve the patient in deciding upon a plan (e.g. options, rationale, values, preferences, concerns)	
28. Explain likely benefits of the option(s) discussed	
29. Explain likely side effects and risks of the option(s) discussed	
30. Provide complete instructions for the plan	
31. Discuss the patient’s ability to follow the plan (e.g. attitude, time, resources)	
32. Discuss the importance of the patient’s role in treatment or prevention	

SEGUE, Set the stage, Elicit information, Give information, Understand the patient’s perspective, and End the encounter.
